# ISSR-Based Genetic Diversity and Structure of *Medicago sativa* L. Populations from the Aras Basin, a Crossroad of Gene Centers

**DOI:** 10.3390/life16010021

**Published:** 2025-12-23

**Authors:** Baris Eren

**Affiliations:** Department of Agricultural Biotechnology, Faculty of Agriculture, Iğdır University, Iğdir 76000, Türkiye; bariseren86@gmail.com

**Keywords:** *M. sativa*, ISSR markers, Aras Basin, population structure, genomic clustering, ecological transition zone

## Abstract

The Aras Basin, located at the intersection of three major gene centers, represents one of the most important transition zones for the evolution of forage legumes. This study evaluates the genetic diversity and population structure of 74 *Medicago sativa* genotypes, including wild populations and commercial cultivars, using ISSR markers. The analysis revealed a broad level of genetic variability, reflecting the adaptive potential of alfalfa in this ecologically heterogeneous region. Population structure analyses consistently separated the germplasm into three genetic clusters, demonstrating clear differentiation between wild accessions and registered varieties. Geographical patterns were also evident, with genotypes from western, central, and eastern subregions forming distinct groups. These results highlight the unique genomic composition of alfalfa in the Aras Basin and demonstrate the value of ISSR markers for characterizing multilayered genetic variation in ecological transition zones. The findings provide a complementary genomic perspective that expands existing knowledge of *M. sativa* diversity and offers useful guidance for breeding programs and genetic resource conservation.

## 1. Introduction

Alfalfa (*Medicago sativa* L.) is one of the world’s most widespread and economically important forage crops, with a cultivation area of approximately 35 million hectares [1,2]. Thanks to its high biomass production capacity, crude protein content ranging from 15–25%, and rich mineral and vitamin content, it is used as a primary feed source in animal nutrition for both dairy and meat production [3]. It also increases soil fertility and reduces chemical fertilizer use by fixing atmospheric nitrogen through Rhizobium species that live symbiotically in its root nodules. Due to these characteristics, alfalfa, often referred to as the “queen of forage plants,” is at the center of sustainable agricultural systems and production models that preserve ecological balance [4].

Alfalfa’s long evolutionary history and broad ecological adaptation ability have led to considerable genetic variation within the species. This variation constitutes an important genetic resource for modern breeding programs. The conservation and characterization of germplasm collections are fundamental steps in developing new varieties that are resistant to biotic and abiotic stresses such as drought, salinity, and diseases [5]. Therefore, local and wild genotypes, in particular, harbor unique alleles that can be transferred to modern varieties as a result of the adaptation mechanisms they have developed [6,7]. Despite the ecological and evolutionary importance of the Aras Basin, no comprehensive ISSR-based evaluation of its wild and cultivated *M. sativa* germplasm has yet been conducted. Previous studies in the region have focused on single marker systems or limited sample sets, leaving a gap in understanding how genomic variability is structured across ecological gradients and between wild and commercial materials [5]. Therefore, the genetic landscape of alfalfa in this biogeographically unique zone remains insufficiently resolved.

Turkey stands out as a strategic biodiversity area for alfalfa due to its location at the intersection of the Transcaucasus, Near East, and Mediterranean gene centers [8]. The Aras Basin is recognized as a major evolutionary transition zone connecting the Irano-Turanian, Mediterranean, and Caucasian gene centers [9,10]. According to Vavilov’s [9] gene center theory, the Aras Basin lies at the intersection of three major gene centers, placing the region in a critical position in terms of agricultural plant evolution. Natural selection processes that have continued for many years in this region have paved the way for the emergence of genotypes adapted to different ecological niches. The Aras Basin, located within the borders of Iğdır province, is at the center of gene flow due to its climatic diversity, microecological conditions, and geostrategic location; therefore, it constitutes a unique resource for researching both genetic diversity and population structure [11].

Taxonomically, alfalfa (*M. sativa* L.) and its cultivated forms belong to the *M. sativa-falcata* species complex, which includes numerous taxa naturally distributed across North Eurasia [12,13]. Due to its economic importance, the *M. sativa-falcata* species complex has long been the focus of botanical, genetic, and agricultural research [14]. Traditional classifications based on flower color, fruit morphology, and pollen characteristics often yield conflicting results due to intense natural hybridization and gene flow [15]. Advances in molecular biotechnology have enabled a reassessment of this species complex and led to the reclassification of many taxa at the subspecies level [16]. Today, the species are classified as six subspecies: *M. sativa* subsp. caerulea, *M. sativa* subsp. falcata, *M. sativa* subsp. ×hemicycla, *M. sativa* subsp. glutinosa, *M. sativa* subsp. sativa, *M. sativa* subsp. ×varia, and *M. sativa* subsp. Glomerata [17]. The *M. sativa-falcata* species complex contains diploid (2n = 2x = 16) and tetraploid (2n = 4x = 32) cytotypes, and there is no reproductive barrier between these cytotypes. *M. sativa* subsp. sativa, the tetraploid form with purple flowers, is the most widely cultivated form worldwide due to its high biomass production and broad agricultural adaptation [18,19]. However, the tetraploid genome structure (2n = 4x = 32) causes a certain level of complexity in classical breeding and molecular genetic analyses [20].

Local genotypes are an important source of variation, particularly in terms of environmental adaptation and stress tolerance [21]. Shaped by long-term natural selection processes and traditional farming practices, these genotypes may contain unique allele combinations that confer resistance to drought, salinity, pests, and diseases [22]. This genetic diversity holds great potential both for the conservation of genetic resources and for the development of new varieties adapted to climate change for use in modern breeding programs [18]. In this context, the molecular characterization of local genotypes is of great importance in understanding the structure of genetic variation and establishing a scientific basis for sustainable alfalfa breeding programs.

Among the methods used in germplasm characterization, molecular marker technologies offer significant advantages because they provide direct information at the DNA level, beyond morphological and agronomic traits. While morphological data can be strongly influenced by environmental factors, molecular markers enable reliable and reproducible results independent of environmental changes [23,24,25]. In this regard, different molecular marker systems such as RAPD, AFLP, SSR, SRAP, SNP, iPBS, and ISSR have been widely used to determine genetic diversity in alfalfa genotypes [4,19,21]. Among these systems, ISSR (Inter-Simple Sequence Repeat) markers stand out due to their high polymorphism rates, rapid applicability, and low costs; they are considered an effective tool for determining genetic diversity and elucidating population structure [11]. Additionally, ISSR markers facilitate the determination of suitable parent selection in breeding programs, enabling more effective use of genetic resources.

The objective of this study was to evaluate the genetic variability and population structure of wild and cultivated *M. sativa* genotypes from the Aras Basin using ISSR markers, and to determine how these patterns correspond to geographical and ecological variation [6,23]. In this study, a total of 74 *M. sativa* L. genotypes were evaluated, including 56 local (wild) alfalfa genotypes collected from the Aras Basin within the borders of Iğdır Province, Turkey, and 18 registered varieties [7]. Inter-Simple Sequence Repeat (ISSR) markers were used to determine genetic diversity and population structure, a method widely applied in alfalfa and related forage species due to its ability to detect multilocus genomic variation [1,3]. The main objective of the study was to reveal the genetic relationships between local germplasm and existing registered varieties, to determine the levels of variation within and between populations, and to evaluate the information obtained as a scientific basis that could be used in future breeding programs, parent selection strategies, and genetic resource conservation efforts [11]. By clarifying the genomic diversity of alfalfa in one of its key evolutionary hotspots, this study provides a needed foundation for future breeding and conservation strategies in the region [8]. Previous studies conducted in Eastern Anatolia have evaluated the genetic diversity of *M. sativa* using iPBS [4] and SCoT markers [5]. While these studies provided valuable insights, they focused on different marker systems and included more limited germplasm sets. The present ISSR-based analysis differs fundamentally from these works by targeting microsatellite-flanking regions, incorporating a broader representation of wild and commercial germplasm, and performing additional analyses such as primer–primer correlations, Jaccard heat maps, and geographical clustering. Therefore, this study offers an independent and complementary genomic perspective on the diversity of alfalfa in the Aras Basin.

Therefore, this study aims to provide the first comprehensive ISSR-based assessment of the genetic diversity and population structure of *M. sativa* in the Aras Basin, a major evolutionary transition zone where three global gene centers intersect. By analyzing both wild germplasm and registered cultivars, we seek to clarify how genetic variation is distributed across ecological subregions and to generate a genomic framework that will support future breeding, parent-selection strategies, and conservation programs.

## 2. Materials and Methods

All procedures followed standard molecular marker protocols and were performed in accordance with established guidelines [23,25,26].

### 2.1. Plant Material

**Figure 1:**
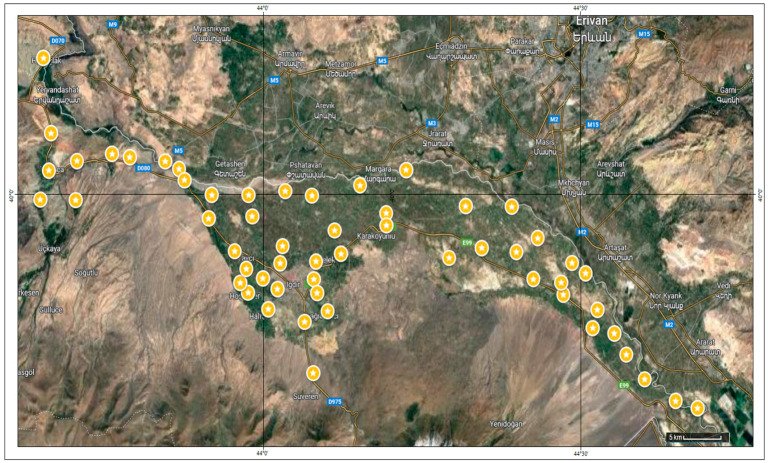
Geographic distribution of alfalfa (*M. sativa* L.) germplasm locations in the Aras basin.

A total of 74 *M. sativa* genotypes were analyzed in this study. Wild materials (*n* = 56) were collected from natural populations across the Aras Basin (Iğdır Province, Türkiye) following institutional collection permits, and GPS coordinates for each sampling site were recorded (Table 1; Figure 1). Field collections were conducted with verbal authorization obtained from the Iğdır Provincial Directorate of Agriculture and Forestry, in accordance with local regulations. GPS coordinates of all sampling locations are provided in Table 1, and each genotype was assigned an accession code (G1–G74). The remaining 18 genotypes consisted of registered commercial varieties obtained from certified seed companies and agricultural research institutes in Türkiye. All accessions were assigned unique codes at the time of sampling and stored under controlled conditions until DNA extraction.

### 2.2. DNA Extraction and Quantification

Genomic DNA was extracted from young leaf tissues using a modified CTAB protocol [27]. DNA quality and concentration were evaluated by agarose gel visualization and spectrophotometric readings, and samples with clear high-molecular-weight bands and A260/280 ratios between 1.8–2.0 were accepted for downstream analyses. All DNA samples were diluted to a working concentration of 20–50 ng µL^−1^ and stored at −20 °C until ISSR amplification.

### 2.3. ISSR Analyses

A set of 16 ISSR primers previously reported to generate clear and reproducible polymorphisms in *M. sativa* was selected for marker amplification. PCR reactions were conducted in a total volume of 20 µL using standard reaction buffer, MgCl_2_, dNTPs, primer, Taq DNA polymerase, and approximately 20 ng of template DNA [4,11]. Amplifications followed a typical ISSR cycling profile consisting of an initial denaturation, 35 cycles of denaturation–annealing–extension, and a final extension step. Annealing temperatures were optimized individually for each primer. PCR products were separated on agarose gels and visualized under UV illumination. All amplifications were performed in duplicate, and only clear, consistently reproducible bands were scored. Faint or ambiguous bands were excluded from the dataset. Band presence was recorded as binary data (1 = band present; 0 = band absent), following a standard scoring threshold.

### 2.4. Data Analysis

**Figure 2:**
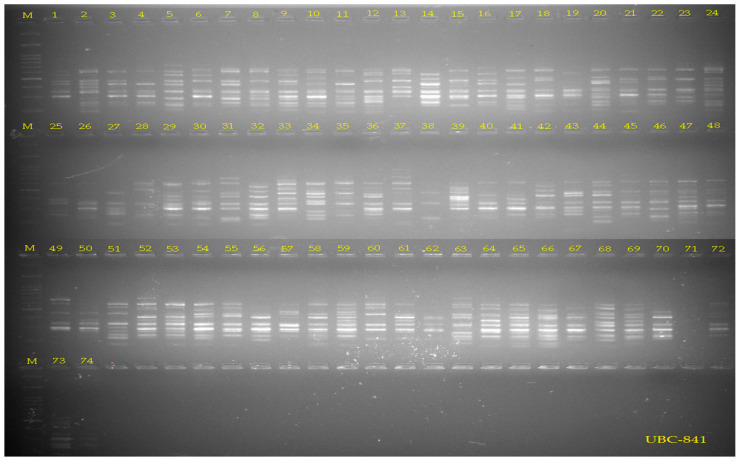
ISSR primer UBC-841 PCR amplification patterns showing polymorphic band profiles among 74 *M. sativa* genotypes on 2% agarose gel.

Bands were scored as binary data (1 = presence, 0 = absence), and only clear, reproducible bands consistently appearing in repeated amplifications were retained for analysis (Figure 2). For the quantitative assessment of genetic diversity, the polymorphism rate (P%), Shannon information index (I), observed and expected allele frequency (Na, Ne), Nei genetic diversity index (He), and polymorphic information content (PIC) were calculated [27,28]. Genetic similarity between genotypes was calculated using the Jaccard similarity coefficient; UPGMA (Unweighted Pair Group Method with Arithmetic Mean) clustering analysis was performed in NTSYSpc v.2.2 software based on the similarity matrix obtained [29]. PCoA (Principal Coordinate Analysis) was performed using the vegan and ggplot2 packages in R software version 4.3.2 (R Core Team, 2024 [30]) to determine the genetic variation and spatial distribution among genotypes. STRUCTURE v.2.3.4 software [31] was used to determine the population structure. STRUCTURE v2.3.4 was run under a mixture model with correlated allele frequencies and the ΔK method indicated a primary peak at K = 3 and a secondary peak at K = 6. The burn-in was set to 50,000 iterations, followed by 100,000 MCMC iterations. K was tested between 1 and 10, with 10 independent runs per K. The most likely K value was determined using the Evanno ΔK method implemented in the independent Structure Selector software (v1.1). Evaluation of the ΔK distribution revealed a major peak at K = 3 and a smaller secondary peak at K = 6, indicating that the primary population structure is best represented by three genetic clusters, while additional sub-structuring appears at higher K values. The analysis was performed under the admixture model and correlated allele frequencies assumption; the most likely number of subpopulations (K) was determined using the Structure Harvester v.0.6.94 tool according to the Evanno et al. [32] method. Since the online Structure Harvester service is no longer operational, ΔK values were computed using Structure Selector [33], which implements the Evanno method for determining the most likely number of clusters. Genetic relationships between ISSR primers and genotypes were also visualized using Pearson correlation matrices and heat maps.

## 3. Results

### 3.1. DNA Polymorphism and Genetic Diversity

ISSR markers produced a high level of polymorphism, revealing substantial genetic variability among the 74 alfalfa genotypes. Across all primers, band numbers and PIC values showed broad variation, demonstrating the strong discriminatory capacity of the marker system (Table 2). The Jaccard similarity coefficients, derived from the full pairwise similarity matrix (Supplementary Table S1), ranged from 0.0147 to 0.4762. Rather than relying on individual numerical values, these patterns collectively indicate that the Aras Basin germplasm harbors high genomic variability. This average value is provided only as a descriptive indicator of primer amplification efficiency; biological interpretation is based on the distribution and variability of band numbers across primers rather than the mean alone.

### 3.2. Genetic Similarity Heat Map Among Primers

**Figure 3:**
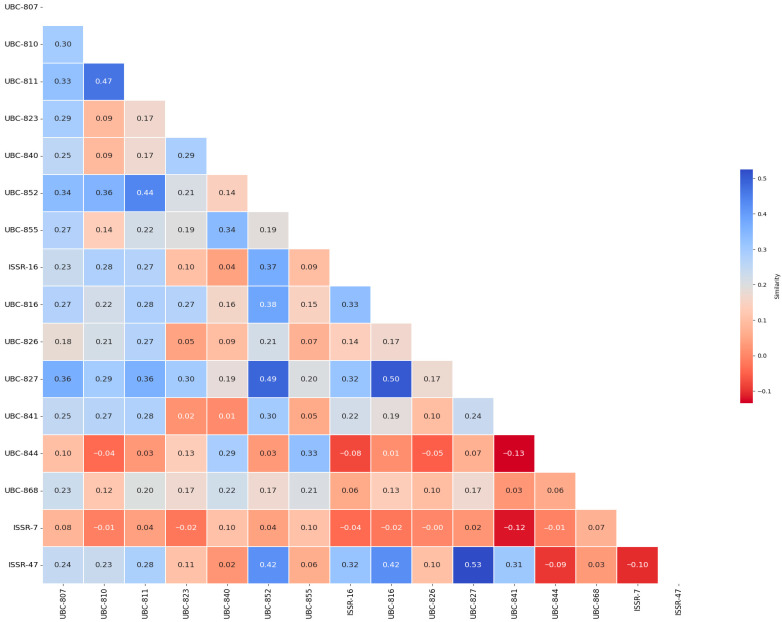
Heat map of correlation similarity coefficients between ISSR primers used in alfalfa genotypes.

Pearson correlation analysis among ISSR markers revealed that relationships among primers were generally weak to moderate (Figure 3). The correlation coefficients ranged from −0.13 to 0.53, indicating a wide range of genetic independence among the markers. The highest positive correlation was found between UBC-827 and ISSR-47 (r = 0.53), while the strongest negative relationship occurred between UBC-844 and ISSR-7 (r = −0.13). These low to moderate correlation values demonstrate that the ISSR primers used target different regions of the genome, thereby providing high discriminatory power for genetic diversity assessment in *M. sativa* genotypes.

### 3.3. Cluster Analysis and Population Structure

**Figure 4:**
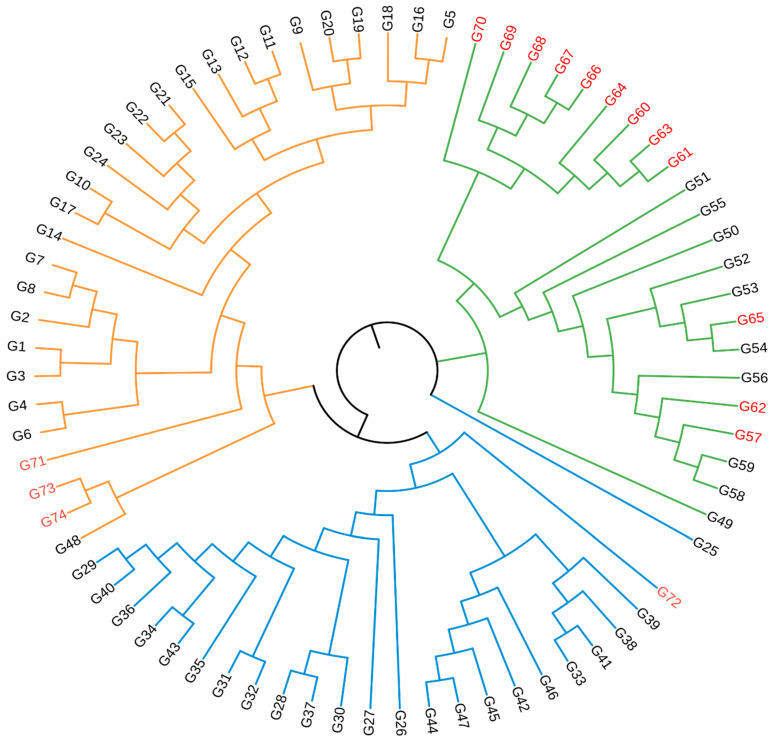
UPGMA dendrogram of alfalfa genotypes. Branches colored in green, blue, and orange correspond to Cluster I, Cluster II, and Cluster III, respectively, as identified by UPGMA clustering. Genotype labels shown in red indicate cultivated varieties, while black labels represent wild genotypes.

The UPGMA dendrogram obtained (Figure 4) showed a distinct genetic structure among genotypes, and the population was divided into three main clusters (Cluster I–Cluster III). The UPGMA dendrogram grouped the genotypes into three main clusters (Figure 4). Cluster I predominantly contained wild genotypes from the western part of the Aras Basin. Cluster II included a mixture of central-region wild genotypes together with several local varieties. Cluster III consisted mainly of commercial cultivars and a few wild accessions collected from the eastern zones. Clusters I–III represent three distinct genetic groupings identified by UPGMA analysis.

### 3.4. Jaccard Similarity Heat Map Among Genotypes

**Figure 5:**
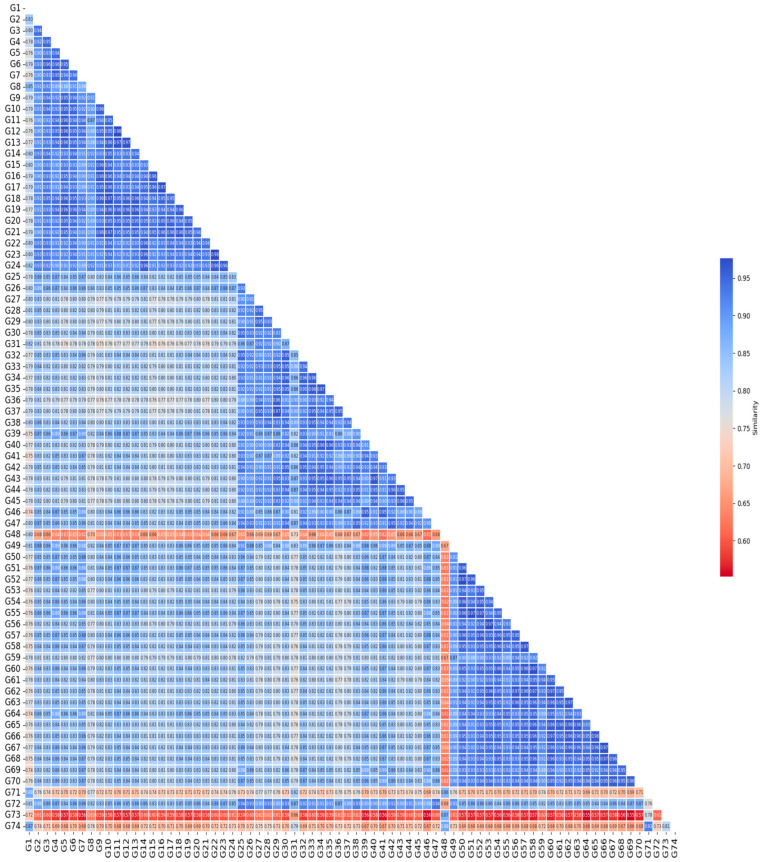
Heat map of Jaccard similarity coefficients between alfalfa (*M. sativa* L.) genotypes.

The heat map based on Jaccard similarity revealed wide genetic divergence, with most genotype pairs showing low similarity values (Figure 5). A small number of genotype pairs exhibited relatively higher similarity, but these were exceptions within a generally heterogeneous dataset. The overall distribution of similarity values—rather than specific pairwise extremes—demonstrates the highly diverse genetic background of the population. These low pairwise similarity values confirm high genetic variability within the studied germplasm. The mean Jaccard coefficient (0.1568) is presented as a descriptive summary; however, biological interpretations are based on the overall distribution and range of genetic similarities rather than the average value itself. This variability refers to differences among individual genotypes and should not be interpreted as population-level genetic differentiation.

### 3.5. Principal Component Analysis (PCoA)

**Figure 6:**
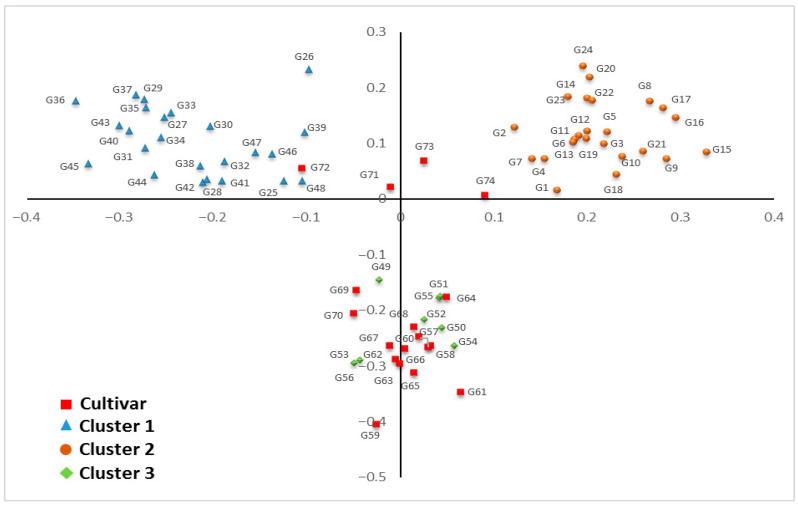
Principal coordinate analysis (PCoA) showing genetic relationships among 74 alfalfa genotypes based on ISSR marker data.

PCoA supported the UPGMA-based structure, separating the genotypes into three distinct groups along the first two coordinates, which together explained a meaningful portion of total molecular variance (Figure 6). The spatial distribution of points on the biplot further illustrates the genetic coherence within clusters and the divergence between them.

### 3.6. STRUCTURE Analysis

**Figure 7:**
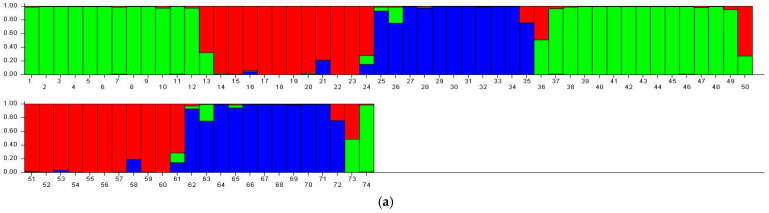

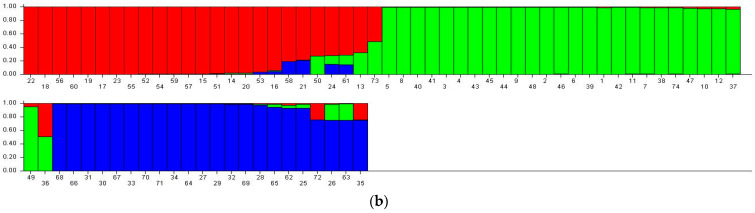
Genetic structure distribution of *M. sativa* genotypes according to STRUCTURE analysis. (**a**) Individual membership ratios arranged according to genotype order; (**b**) Subpopulation structure arranged according to clustering order. Red (Q1), blue (Q2), and green (Q3) colors represent the three main genetic populations.

**Figure 8:**
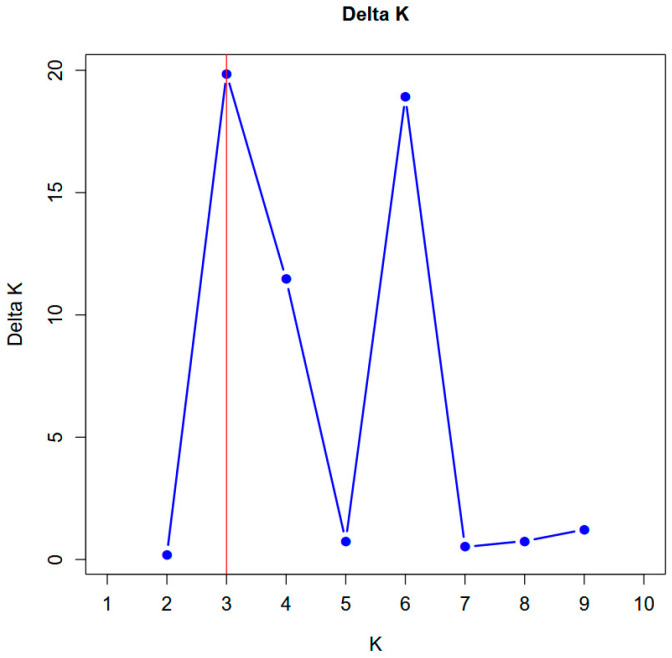
ΔK values used to identify the most likely number of genetic clusters (K) based on STRUCTURE analysis.

STRUCTURE analysis (admixture model, correlated allele frequencies) identified K = 3 as the most likely number of genetic groups based on Evanno’s ΔK criterion. Replicate runs produced consistent clustering patterns, which aligned well with both UPGMA and PCoA. Most individuals showed high membership coefficients, while several accessions displayed admixture profiles, suggesting ongoing gene flow within the region (Figure 7 and Figure 8).

### 3.7. Distribution of Genetic Diversity

Based on marker performance, cluster separation, and similarity distributions, Cluster II appeared to exhibit the highest internal genetic variability, likely due to its inclusion of both wild genotypes and local cultivars. In contrast, Cluster III—dominated by commercial varieties—showed the most genetic uniformity.

## 4. Discussion

The genetic diversity and population structure of *M. sativa* genotypes collected from the Aras Basin revealed extensive genomic variation based on ISSR markers, reflecting the high adaptive capacity and evolutionary potential of alfalfa populations in this ecogeographically heterogeneous region [6]. The wide range of polymorphisms and low similarity coefficients indicate a heterogeneous germplasm structure characterized by broad allelic variation and distinct genetic groupings, consistent with earlier ISSR-based studies on Turkish and Iranian alfalfa populations reporting strong intraspecific polymorphism [7,39].

The high diversity observed in the Aras Basin is biologically expected given the region’s unique biogeographic context [40,41,42]. Located at the intersection of the Irano-Turanian, Mediterranean, and Caucasian gene centers, the basin functions as a transition zone where contrasting climatic regimes, elevational gradients, and microecological niches converge. Geographic barriers such as fragmented valleys and foothill systems promote partial isolation among local *Medicago* populations, while long-term grazing pressure and land-use variability contribute to both differentiation and admixture [43]. These ecological factors help explain the mosaic genetic structure observed in the present dataset [40,41].

ISSR markers produced a high proportion of polymorphic bands, indicating substantial genomic variability among genotypes. Similar confirmations from Chinese and Iranian *M. sativa* populations show that ISSR markers effectively capture genomic responses to local environmental heterogeneity [44,45]. While ISSR-based similarity matrices reflect genetic variability among individual genotypes, the UPGMA, PCoA, and STRUCTURE analyses specifically demonstrate genetic differentiation at the population-group level. Together, these independent approaches converge on three major subgroupings, strengthening the reliability of the inferred population structure. Comparisons with previous SCoT, iPBS, ISSR, and SSR studies conducted in Turkey, Iran, and Central Asia indicate that the Aras Basin consistently emerges as a hotspot of alfalfa diversity [21,46,47]. Although earlier studies documented substantial intraspecific variability, the present ISSR-based results expand this perspective by revealing independent genomic dimensions and clearer subgroup formations unique to microsatellite-flanking regions. These findings support the view that Eastern Anatolian alfalfa germplasm possesses multilayered genetic complexity rather than a simple east–west gradient of variation.

STRUCTURE analysis further highlighted meaningful biological patterns. The K = 3 solution indicates the presence of western wild populations, central mixed populations, and a third cluster enriched with cultivated varieties and eastern genotypes. This partitioning correlates closely with ecological subdivisions and suggests region-specific evolutionary histories shaped by topography, gene-flow corridors, and historical land-use patterns. Similar findings were reported by Şakiroğlu, Doyle, and Brummer [21] using SSR markers, who also observed elevated gene flow in areas near river valleys—consistent with the low similarity rates and recombination signals identified in the present study. Although a secondary ΔK peak appeared at K = 6, such higher-order peaks are common in hierarchical population structures and typically represent sub-structuring rather than primary clusters [32]. Therefore, K = 3 was considered biologically meaningful and is consistent with geographical patterns in the Aras Basin.

The identified genetic clusters have important implications for breeding and conservation. Genotypes showing strong divergence from cultivated varieties may serve as valuable donors of adaptive alleles for improving traits such as drought tolerance, salinity tolerance, and biomass productivity [20,48,49]. Conversely, clusters with mixed ancestry highlight regions of active gene flow and ongoing evolutionary processes, which are essential for maintaining adaptive potential and sustaining genetic resources [21,50]. The differentiation among groups appears closely associated with topographic heterogeneity and microclimatic variation, a relationship supported by broader forage crop studies from Eastern Anatolia [51]. Accordingly, genotypes originating from the Aras Basin may hold strategic importance for future genome-assisted selection and marker-based breeding programs.

Unlike previous studies conducted in the region, the present research integrates a larger germplasm set (74 genotypes), includes a broader representation of commercial cultivars, and applies a more comprehensive analytical framework incorporating STRUCTURE, PCoA, primer–primer correlations, and genotype-level similarity heat maps [4,5]. These analyses reveal previously unreported genetic subgroupings, novel admixture patterns in cultivated varieties, and marker-specific genomic independence among ISSR loci [1,3]. Therefore, the findings provide a novel and expanded genomic perspective on *M. sativa* populations in the Aras Basin.

Nevertheless, the study has certain limitations. ISSR markers are dominant and cannot distinguish heterozygous from homozygous loci. Sampling was restricted to a specific geographic area, and no phenotypic or environmental variables were included. Broader sampling, co-dominant marker systems, and phenotypic characterization could enhance resolution in future research. Overall, the genetic patterns revealed here underscore the Aras Basin’s importance as a reservoir of alfalfa diversity. The convergence of evidence across multiple analytical approaches highlights the basin’s evolutionary significance and agronomic potential, providing a valuable foundation for breeding programs, conservation planning, and future genomic investigations.

## 5. Conclusions

This study provides a comprehensive genomic assessment of *M. sativa* populations from the Aras Basin, a region where three major gene centers intersect and generate exceptional evolutionary and ecological complexity. ISSR-based analyses revealed multilayered genetic variation across wild and cultivated genotypes, demonstrating that the basin hosts one of the most heterogeneous alfalfa gene pools in Eastern Anatolia. The convergence of STRUCTURE, UPGMA, and PCoA results indicates the presence of three major genetic groups shaped by geographic gradients, microecological heterogeneity, and ongoing gene flow. These patterns confirm that the Aras Basin functions not merely as a cultivation area but as a dynamic evolutionary corridor that maintains high allelic richness. The findings have practical implications for breeding and conservation. Genotypes exhibiting strong divergence from commercial cultivars represent valuable genetic resources for improving stress tolerance, biomass productivity, and environmental resilience. Conversely, admixed genotypes highlight regions where natural gene flow remains active, offering potential parent material for population improvement and genomic selection programs. Compared to earlier studies, this research expands the existing knowledge by incorporating a larger and more diverse germplasm set, applying a broader analytical framework, and revealing previously unreported subgroup formations and marker-specific genomic patterns. These contributions provide a more refined understanding of the evolutionary structure of alfalfa in a key geographic region.

Future research integrating morphological traits, adaptive phenotypes, and broader geographic sampling—including Western and Central Anatolia—will further strengthen our understanding of alfalfa diversity and enhance the applicability of these molecular insights.

Overall, our findings demonstrate that the Aras Basin represents a strategically important reservoir of alfalfa genetic diversity, making it a priority landscape for future genomic research, germplasm conservation, and cultivar development.

## Figures and Tables

**Table 1:** Characteristics of alfalfa genotypes and varieties used in the study.

No.	Location	Trait	No.	Province		Trait
G1	39°46′59.5″ N 44°40′54.1″ E	Wild	G38	40°00′20.5″ N 43°58′24.3″ E		Wild
G2	39°47′26.9″ N 44°38′50.2″ E	Wild	G39	39°56′49.5″ N 43°57′04.9″ E		Wild
G3	39°48′48.5″ N 44°35′53.5″ E	Wild	G40	39°54′13.7″ N 43°58′21.5″ E		Wild
G4	39°50′21.3″ N 44°34′09.1″ E	Wild	G41	39°55′43.3″ N 43°58′10.9″ E		Wild
G5	39°51′40.5″ N 44°33′02.0″ E	Wild	G42	39°54′50.5″ N 43°57′36.6″ E		Wild
G6	39°53′09.3″ N 44°31′25.0″ E	Wild	G43	39°58′59.9″ N 43°58′44.5″ E		Wild
G7	39°52′00.7″ N 44°30′58.9″ E	Wild	G44	40°00′34.7″ N 44°01′52.4″ E		Wild
G8	39°55′26.4″ N 44°30′18.3″ E	Wild	G45	39°57′08.9″ N 44°01′37.4″ E		Wild
G9	39°54′06.3″ N 44°28′11.7″ E	Wild	G46	39°53′11.6″ N 44°00′15.9″ E		Wild
G10	39°56′05.5″ N 44°29′01.0″ E	Wild	G47	39°55′07.3″ N 43°59′45.4″ E		Wild
G11	39°54′51.1″ N 44°27′59.6″ E	Wild	G48	39°52′24.2″ N 44°03′42.2″ E		Wild
G12	39°57′38.7″ N 44°25′46.1″ E	Wild	G49	39°55′04.3″ N 44°04′34.3″ E		Wild
G13	39°55′04.9″ N 44°25′21.9″ E	Wild	G50	39°49′11.5″ N 44°04′31.4″ E		Wild
G14	39°56′46.3″ N 44°23′45.6″ E	Wild	G51	39°53′04.1″ N 44°05′53.1″ E		Wild
G15	39°59′36.9″ N 44°23′19.1″ E	Wild	G52	39°56′11.0″ N 44°04′46.7″ E		Wild
G16	39°57′01.5″ N 44°20′28.7″ E	Wild	G53	39°54′12.4″ N 44°04′53.6″ E		Wild
G17	39°59′38.7″ N 44°18′57.2″ E	Wild	G54	39°56′04.1″ N 44°01′23.4″ E		Wild
G18	39°56′24.2″ N 44°17′22.7″ E	Wild	G55	39°54′27.4″ N 44°01′07.6″ E		Wild
G19	40°01′54.2″ N 44°13′18.9″ E	Wild	G56	39°56′39.7″ N 44°07′08.9″ E		Wild
G20	39°59′12.2″ N 44°11′25.4″ E	Wild	G57	Sunter	Mutlu Seed Industry and Trade Co., Ltd. Konya/Türkiye	
G21	39°58′26.5″ N 44°11′26.9″ E	Wild	G58	Kayseri	Local genotype, Kayseri/Türkiye	
G22	40°00′56.6″ N 44°08′56.6″ E	Wild	G59	Magna-601	Biotek Seed Agri. Prod. Ind. & Trade Inc. Konya/Türkiye	
G23	40°00′18.4″ N 44°04′24.1″ E	Wild	G60	La Torre	Maro Agri. Constr. Trade & Ind. Inc. Ankara/Türkiye	
G24	39°58′07.2″ N 44°06′32.6″ E	Wild	G61	Savaş	East Anatolian Agricultural Research Inst. Erzurum/Türkiye	
G25	40°08′54.2″ N 43°38′58.1″ E	Wild	G62	Q. Neobi	NEOBI Seed Inc. İzmir/Türkiye	
G26	40°04′12.6″ N 43°39′41.4″ E	Wild	G63	May İside	May-Agro Seed Co. Bursa/Türkiye	
G27	40°01′52.3″ N 43°39′29.3″ E	Wild	G64	La Bella	Samen-Unternehmung C. Böhrer Austrian	
G28	40°02′28.1″ N 43°42′08.1″ E	Wild	G65	Magnum	Biotek Seed Agri. Prod. Ind. & Trade Konya/Türkiye	
G29	40°00′03.2″ N 43°38′40.6″ E	Wild	G66	Prosementi	Tasaco Agriculture Industry and Trade Inc. Antalya/Türkiye	
G30	40°00′01.5″ N 43°42′01.5″ E	Wild	G67	Gea	Maro Agri. Constr. Trade & Ind. Inc. Ankara/Türkiye	
G31	40°02′54.6″ N 43°45′28.2″ E	Wild	G68	Elçi	Ankara University Faculty of Agriculture, Ankara/Türkiye	
G32	40°02′42.2″ N 43°47′09.0″ E	Wild	G69	Plato	Kazak Agri. Constr. & Transport Ind. & Trade Inc. Ankara/Türkiye	
G33	40°02′26.7″ N 43°50′30.6″ E	Wild	G70	Giulia	Mutlu Seed Industry and Trade Co., Ltd. Konya/Türkiye	
G34	40°01′58.5″ N 43°51′49.2″ E	Wild	G71	Emiliana	Palmiye Seed Agri. Ind. & Trade Co., Ltd. İzmir/ Türkiye	
G35	39°58′52.5″ N 43°54′37.4″ E	Wild	G72	Ezzelina	Alfa Seed Agri-Food-Const-Live. Trade Ltd. Larissa/Greece	
G36	40°01′16.8″ N 43°52′20.0″ E	Wild	G73	Bilensoy-80	Field Crops Central Research Institute, Ankara/Türkiye	
G37	40°00′21.1″ N 43°54′54.6″ E	Wild	G74	Gacer	Iğdır local genotype, Iğdır/Türkiye	

**Table 2:** Some polymorphism parameters of the 16 ISSR markers used in the characterization of 74 *M. sativa* genotypes.

No.	Marker	Primer Seq.	AB	PB	%P	PIC	I	Ne	Rp
1	UBC-807	(AG)_8_ T	14	14	100	0.277	0.630	1.432	0.620
2	UBC-810	(CA)_8_ T	10	10	100	0.334	0.727	1.550	0.535
3	UBC-811	(GA)_8_ C	8	8	100	0.206	0.503	1.287	0.750
4	UBC-823	(GA)_8_ C	7	7	100	0.141	0.374	1.175	0.842
5	UBC-840	(GA)_8_ TT	6	6	100	0.244	0.549	1.385	0.676
6	UBC-852	(TC)_8_ AA	12	12	100	0.237	0.552	1.358	0.691
7	UBC-855	(AC)_8_ YT	8	8	100	0.218	0.529	1.298	0.740
8	ISSR-16	(GTGC)_4_	8	8	100	0.200	0.476	1.319	0.720
9	UBC-816	(GA)_8_ T3	17	17	100	0.230	0.542	1.337	0.711
10	UBC-826	(GA)_8_ C3	17	17	100	0.260	0.606	1.387	0.661
11	UBC-827	(CA)_8_ G	17	17	100	0.236	0.548	1.361	0.688
12	UBC-841	(GA)_8_ YC	23	23	100	0.293	0.651	1.473	0.591
13	UBC-844	(CT)_8_ AC	6	6	100	0.205	0.483	1.314	0.730
14	UBC-868	(GAA)_6_	12	12	100	0.269	0.609	1.418	0.644
15	ISSR-7	(TC)_8_ C	15	15	100	0.222	0.541	1.302	0.735
16	ISSR-47	(AG)_8_ Y	33	33	100	0.285	0.640	1.457	0.609
	Mean		213	213		0.241	0.560	1.366	0.684

AB: Amplified Bands; PB: Polymorphic Bands; %P: Percentage of Polymorphism [34]; PIC: Polymorphic Information Content [35]; I: Shannon’s Information Index [36]; Ne: Effective number of alleles [37]; Rp: Resolving Power [38].

## Data Availability

The data presented in this study are available on request from the corresponding author. The data are not publicly available due to institutional data policy and privacy restrictions.

## Supplementary Materials

The following supporting information can be downloaded at: https://www.mdpi.com/article/10.3390/life16010021/s1, Table S1: Pairwise Jaccard Coefficients Among Medicago sativa Genotypes (ISSR Markers).

## Abbreviations

The following abbreviations are used in this manuscript:

ISSR	Inter-Simple Sequence Repeat
PCR	Polymerase Chain Reaction
PCoA	Principal Coordinate Analysis
UPGMA	Unweighted Pair Group Method with Arithmetic Mean
PIC	Polymorphism Information Content
CTAB	Cetyl Trimethylammonium Bromide
He	Nei’s Genetic Diversity (Expected Heterozygosity)
Ne	Effective Number of Alleles
Rp	Resolving Power
ΔK	Delta K (Evanno method index for STRUCTURE analysis)
